# Diaper Duty: a CAUTI Reduction Initiative

**DOI:** 10.1097/pq9.0000000000000684

**Published:** 2024-02-21

**Authors:** Hadassah L. Little

**Affiliations:** 1 From the Shawn Jenkins Children’s Hospital, Medical University of South Carolina, Charleston, SC.

## Background:

After a spike in catheter-associated urinary tract infections (CAUTIs) in 2021, this hospital experienced a significant rate increase from 0 to 3.094 CAUTIs per 1,000 catheter days.

## Objective:

The goal was to identify the cause of these infections and decrease the rate of CAUTIs.

## Methods:

At the end of 2021, a common cause analysis was performed through retrospective review of the electronic medical record for each patient who acquired a CAUTI between 2019 and 2021. This review revealed that all infections during this time were caused by intestinal organisms. It was also discovered that each of these patients had documentation indicating diapers were being used while they had an indwelling Foley catheter in place. The plan-do-study-act model was used to evaluate the theory that avoiding the use of diapers in patients with Foleys would reduce the rate of CAUTIs. Compliance with this practice change was monitored via k-card observations and weekly rounding on patients with indwelling Foley catheters. The rounding was facilitated by both clinical and quality leaders and was performed at the bedside to provide education and uncover barriers. Additionally, a new perineal care product was purchased with the goal of standardizing Foley care. The product is intended solely for Foley care and includes clear directions on the packaging.

## Results:

Following these interventions this hospital experienced a significant decrease in CAUTIs. When comparing events from 2022 to 2021, there was a 75% reduction in CAUTIs, and those infections that did occur in 2022 were not caused by intestinal organisms. The rate of CAUTIs has also decreased back to 0 CAUTIs per 1,000 catheter days.

## Conclusions:

The use of diapers on patients with indwelling Foley catheters may result in directing stool toward the catheter, delaying the recognition of the need for Foley care, and creating a bacteria friendly environment near a device that is at risk for infection. The outcomes related to this initiative may indicate that avoiding the use of diapers for patients with Foleys reduces the risk for CAUTIs, especially those caused by intestinal organisms.

